# Long time frames to detect the impact of changing COVID-19 measures, Canada, March to July 2020

**DOI:** 10.2807/1560-7917.ES.2021.26.40.2001204

**Published:** 2021-10-07

**Authors:** Jessica E Stockdale, Renny Doig, Joosung Min, Nicola Mulberry, Liangliang Wang, Lloyd T Elliott, Caroline Colijn

**Affiliations:** 1 Department of Mathematics, Simon Fraser University, Burnaby BC, Canada; 2 Department of Statistics and Actuarial Science, Simon Fraser University, Burnaby BC, Canada

**Keywords:** SARS-CoV-2, COVID-19, physical distancing, control, Canada, modelling

## Abstract

**Background:**

Many countries have implemented population-wide interventions to control COVID-19, with varying extent and success. Many jurisdictions have moved to relax measures, while others have intensified efforts to reduce transmission.

**Aim:**

We aimed to determine the time frame between a population-level change in COVID-19 measures and its impact on the number of cases.

**Methods:**

We examined how long it takes for there to be a substantial difference between the number of cases that occur following a change in COVID-19 physical distancing measures and those that would have occurred at baseline. We then examined how long it takes to observe this difference, given delays and noise in reported cases. We used a susceptible-exposed-infectious-removed (SEIR)-type model and publicly available data from British Columbia, Canada, collected between March and July 2020.

**Results:**

It takes 10 days or more before we expect a substantial difference in the number of cases following a change in COVID-19 control measures, but 20–26 days to detect the impact of the change in reported data. The time frames are longer for smaller changes in control measures and are impacted by testing and reporting processes, with delays reaching ≥ 30 days.

**Conclusion:**

The time until a change in control measures has an observed impact is longer than the mean incubation period of COVID-19 and the commonly used 14-day time period. Policymakers and practitioners should consider this when assessing the impact of policy changes. Rapid, consistent and real-time COVID-19 surveillance is important to minimise these time frames.

## Introduction

In response to the coronavirus disease (COVID-19) pandemic, many countries have implemented large-scale non-pharmaceutical interventions (NPI), with a particular focus on physical distancing measures. The details of physical distancing vary substantially between and within countries. Differences in the severity and timeliness of the response, different patterns of social contact within a community and varying public compliance modulate the effect of distancing measures on a region’s epidemic trajectory. Accordingly, countries experiencing increasing case counts have considered, and need to continue considering, what degree of distancing measures to implement in order to reduce COVID-19 spread while minimising negative effects, such as adverse health and economic impacts. Understanding the possible trajectories that may arise from changing physical distancing measures is crucial, as is consideration of the timescale of such trajectory changes to ensure that they result in the desired impact within the expected time frame. Quantifying the timescale of such changes can contribute to, for example, healthcare capacity forecasting and delivery of timely advice and instructions to communities.

A number of recent studies have examined the effectiveness of government measures on the spread of COVID-19 [1-8]. One approach has been to retrospectively compare observed data to baseline model output [2,3]. Several studies have focused on estimating changes in the effective reproduction number over time [4,9,10]. Similarly, Anderson et al. directly estimated the impact of control measures on COVID-19 transmission patterns using a Bayesian model with explicit physical distancing [1]. However, these studies do not focus on reporting the predicted time until a given amount of change occurs across many simulations of future case counts. Instead, they compare simulations to a constant baseline, or report quantitative measures on only a few simulations.

We used a likelihood-based approach to determine when we may expect to see the effects of implementing or relaxing physical distancing measures using case count data. This is in contrast to the work described above which focuses on quantifying the effect of such measures. We used a susceptible-exposed-infectious-removed (SEIR)-type model to simulate scenarios over a broad range of simulation parameters. We considered the impact of data collection delays and inconsistencies on the time to detect a change in distancing and applied our methods to publicly available case count data from British Columbia (BC), Canada. We focused on the first implementation of physical distancing measures between March and April 2020 and the first relaxation of measures in between May and July 2020. As regions adjust their measures and mandates, particularly in response to COVID-19 vaccination uptake and emergence of new variants, it is crucial to understand the time frame over which we would expect to see the impact of such changes in order to properly assess their effects.

## Methods

**Figure 1:**
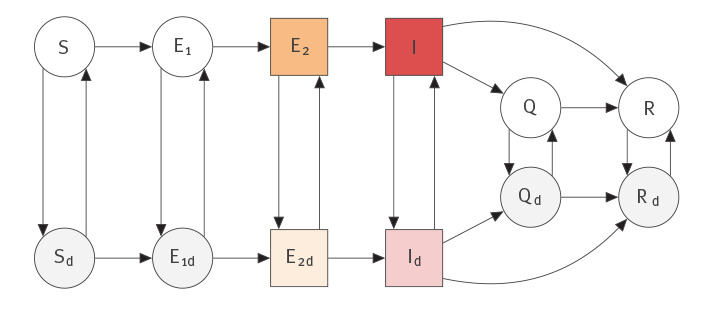
Schematic diagram of the compartmental model for COVID-19 transmission incorporating physical distancing

We used a deterministic SEIR-type model, a compartmental model describing susceptible, exposed, infectious and removed individuals, first developed by Anderson et al. in 2020 [1]. We fitted the model to data on daily case counts and incorporated knowledge of the incubation period and duration of the infectious period in the model. The model includes a fixed proportion of the population who are willing and able to practise physical distancing, although individuals can move between distancing and non-distancing modes. A schematic diagram for this model is shown in Figure 1.

Compartments are: susceptible to the virus (*S*); exposed (*E*_1_); exposed, pre-symptomatic, and infectious (*E*_2_); symptomatic and infectious (*I*); quarantined (*Q*); and removed (recovered or deceased; *R*). There are analogous states for individuals practising physical distancing (bottom row). An individual in state *X* can begin distancing and move to the corresponding distanced state *X_d_* at rate *u_d_*. The reverse transition occurs at rate *u_r_*. The model quickly settles on a fraction *e* = *u_d_*/(*u_d_* + *u_r_*) participating in distancing, and dynamics depend on this fraction, rather than on the rates *u_d_* and *u_r_* The red and tinted red squares represent the compartments that we identify as active cases. The orange/orange tinted, and red/red tinted compartments indicate infectious cases.

This schematic diagram was adapted from Anderson et al. [1].

The strength of physical distancing is represented by parameter 0 ≤ *ƒ*(*t*) ≤ 1, *ƒ*(*t*) = 1 indicating no physical distancing and *ƒ*(*t*) = 0 indicating a complete avoidance of all contacts. Distancing individuals transmit infection at a reduced rate and are less likely to encounter others. We modelled the introduction and relaxation of distancing as follows:

$$f\left(t\right)=\{\begin{array}{ll} f_{0} & \text{before distancing is enacted}\\ f_{1} & \text{when distancing measures are in place}\\ f_{2} & \text{after some relaxation of distancing}\text{.} \end{array}$$

To relate observed daily case numbers to the underlying transmission model, we took the mean number of new cases reported on day *t*, *μ_t_* to be a weighted sum of those who became symptomatic at some time *s* days ago:

$$\mu_{t}=\psi_{t}\overset{M}{\underset{0}{\int }}k_{2}\left(E_{2}\left(t-s\right)+E_{2d}\left(t-s\right)\right)w_{c}\left(s\right)\text{d}s.$$

Here *w_c_* represents the distribution of delay between symptom onset and a reported positive test, *ψ_t_* is the fraction of eligible cases on day *t* that will be tested and reported, and *M* represents the maximum delay (here, twice the mean delay of 8.78 days, see Supplementary Table S1). We sought to determine (i) how soon changes in distancing measures may cause substantial changes in the number of prevalent cases; and (ii) how soon we may be able to estimate the strength of these measures given uncertainty about the trajectory and delays and noise in reporting. We used a negative binomial likelihood for the observational model with mean *µ_t_* and dispersion parameter φ, NB(C_t_ │ μ_t_, φ) as in [1], to write the likelihood of the data C_t_ on day *t* given the model parameters, where dispersion φ accounts for sources of variability in observation: this may include, for example, test-seeking behaviour, lag times, and test availability/processing. This model framework has been demonstrated to capture population-scale dynamics of COVID-19 case counts in BC as well as other provinces, states and countries [1,11].

We introduced a method for determining the first date at which the prevalence in two model scenarios (a change in physical distancing vs baseline) begin to diverge. We computed the empirical probability that there is a difference between the two models, according to the distributions of prevalence they imply. These distributions are obtained by considering uncertainty in the basic reproduction number *R*_0,_ which captures uncertainty in transmission and duration of infection. In this model

$$R_{0}=\beta \left(\frac{1}{q+\gamma }+\frac{1}{k_{2}}\right)$$

in the absence of distancing (i.e. when *ƒ* = 1), where *β* is the infection rate, *q* is the quarantine rate, *γ* is the recovery rate and *k*_2_ is the rate of moving from the *E*_2_ to *I* compartment (i.e. 1/*k*_2_ is the mean length of the pre-symptomatic infectious period). Given information about the duration, the transmission parameter *β* can simply be scaled in order to match the exponential growth rate for the early stage of the epidemic. Ordinary differential equations (ODEs) which describe the compartmental model are simulated numerically, drawing *R*_0_ from the prior and keeping other parameters fixed. This procedure induces a distribution over the case counts seen on each day. To compute the empirical probability that a substantial difference occurs on a given day after introducing distancing measures, we considered the proportion of simulated samples for which the model with physical distancing shows fewer active cases (prevalent, symptomatic) than the non-distancing model by at least 10. Alternatively, if distancing measures are being relaxed, prevalent cases would increase, and the comparison is reversed.

We computed the above empirical probability daily after a change in distancing. The first day on which it is at least 0.95 is the ‘days until threshold’ i.e. our estimate for the first day at which the effect of modifying physical distancing is substantial. We compared several strengths of change in physical distancing, and for each we considered 100 replicates, each with a value of *R*_0_ drawn from the prior. We performed an additional analysis in which the active cases threshold was varied. In BC, 10 cases corresponded to approximately 5% of the incident cases reported on the day distancing was introduced; the threshold could be modified for other jurisdictions.

In the second part of the analysis, we explored the time it would take to observe such differences in reported case counts, taking into account observation noise and delay. The negative binomial likelihood expression (fully detailed in the Supplementary material) relates predicted case counts from the SEIR-type model to reported case counts, corrected for the delay between symptom onset and reporting. This likelihood, written as a function of a given strength of physical distancing *f_x_*, (e.g. *f*_1_ or *f*_2_) may be maximised to find *f ^n^*_MLE_ the maximum likelihood estimate (MLE) of *f_x_* on day *n*. Estimation of *f ^n^*_MLE_ uses data from the start of the outbreak up to and including day *n*. As well as delays between new cases being infected and becoming symptomatic, and becoming symptomatic and being reported, noise in daily case counts introduces considerable variation in the day-by-day MLEs, particularly in the days following a change in distancing. We introduced a stopping rule in which an estimate *f ^n^*_MLE_ is ‘accepted’ once the MLE changes by less than 5% over a 3-day period.

We used the daily MLE approach to estimate the value of *ƒ*_1_ in the period following the introduction of distancing recommendations in BC (18 March 2020), using daily case counts reported between 1 March and 22 April 2020. We found a credible band by simulating the compartmental model with 50 different values of the baseline *R*_0_ parameter, sampled from a normal distribution with mean *R*_0_ = 2.57 as estimated by a pre-distancing model fit and with standard deviation 0.05. We performed a similar analysis to estimate the value of *f*_2_ in the period following the relaxation of distancing recommendations, using case counts reported between 1 May and 1 July 2020. We used 17 May as a start day of the relaxation of distancing recommendations, coinciding with the holiday weekend on which BC ended Phase 1 of its COVID-19 restrictions [12]. For credible bands, we increased the standard deviation to 0.2 to obtain a similar level of variation compared with the introduction-of-distancing estimation. To explore this further, we also simulated data in which distancing was relaxed to 50%, 65%, and 90% of the normal (unrelaxed) level, under the assumption that the case count observation noise and delay remained as during March and April in BC (φ = 5, delay shape 9.85, delay scale 1.73 [1]), and found the time taken to accept the MLE of *f*_2_.

We investigated the impact of the noise in case count reporting on the time to detect a change in the strength of physical distancing by simulating outbreaks in which we varied the observation dispersion parameter φ. We controlled the variance in the number of daily reported cases: note that this is not the same as the dispersion parameter about *R*_0,_ commonly referred to as *k.* We also simulated outbreaks in which we varied the shape and scale parameters of the onset-to-reporting Weibull distribution, to explore the impact of this delay. We then performed the same daily MLE estimation procedure on each of these simulated time series.

A full description of the model, likelihood and methods is available in Supplement S1. Values of the model parameters for our analysis using BC data are available in Supplementary Table S1. All statistical analysis was performed in R software version 4.0.3 (R Foundation, Vienna, Austria), and datasets and R code are available on GitHub under an open source license [13].

### Ethical statement

All data were collected from publicly available sources; therefore, ethical approval was not required.

## Results

### Time for a change in COVID-19 measures to impact cases and reported case counts

**Figure 2:**
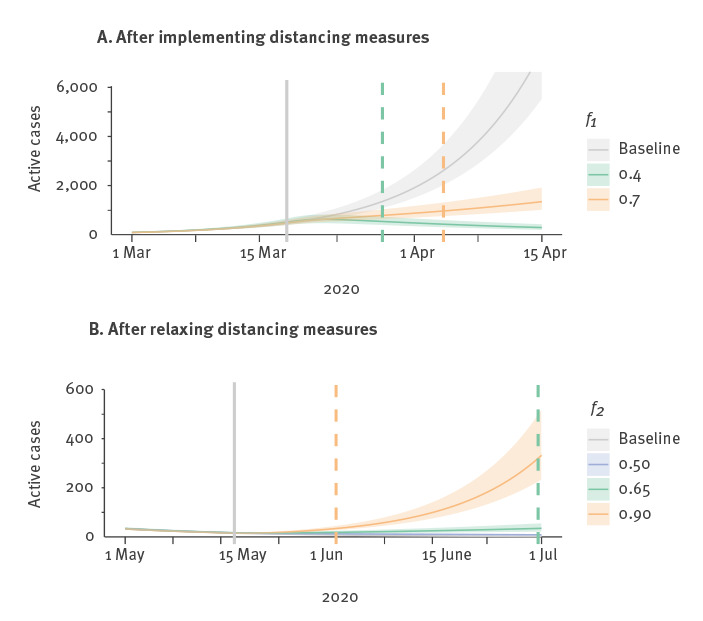
Projected active cases (*I* + *I_d_*) after a change in physical distancing, British Columbia, Canada, March–July 2020

We found that it took 10–17 days (18 March until 28 March or 4 April) before there was a substantial difference between the baseline trajectory (no distancing) and a trajectory with distancing measures of *f*_1_ = 0.4, 0.7 respectively. However, it took 26 days to observe the difference and estimate the strength of distancing using reported data. The situation is similar for relaxing measures: it took 44 days until a substantial difference arose after relaxing distancing from *f*_1_ = 0.36 to *f*_2_ = 0.65, and 20 days to detect such a difference using observed cases. If more drastic relaxation of measures takes place, from *f*_1_ = 0.36 to *f*_2_ = 0.9, a substantial difference in the model trajectories occurs over a much shorter time frame i.e. 14 days. Figure 2 shows declining case trajectories when distancing is introduced, and inclining ones when distancing is relaxed, and illustrates the dependence of the timing on the severity of the change. When the change was weak (e.g. a weak relaxation from *f*_1_ = 0.36 to *f*_2_ = 0.5) there was no discernible difference between the trajectories before 1 July (45 days).

Vertical dashed lines indicate when there is a 0.95 probability of a substantial difference of 10 active cases between the baseline and the altered trajectory.

A. Three models are considered: a baseline model (grey) in which physical distancing is not introduced, and two models in which the amount of physical distancing is modified to be *f*_1_ = 0.7 (orange line), or *f*_1_ = 0.4 (green line). For *f*_1_ = 0.4 a significant change is identified in the model after 10 days, and for *f*_1_ = 0.7 a significant change is identified after 17 days.

B. Three levels of relaxation are considered corresponding to *f*_2_ = 0.5, *f*_2_ = 0.65, and *f*_2_ = 0.9. For the relaxation level *f*_2_ = 0.5, no difference is seen for the period under question (the curves for the *f*_2_ = 0.5 and baseline conditions broadly overlap). For *f*_2_ = 0.5 and *f*_2_ = 0.9, significant changes are seen after 44 and 14 days, respectively.

### Impact of parameter choices on model projections

**Figure 3:**
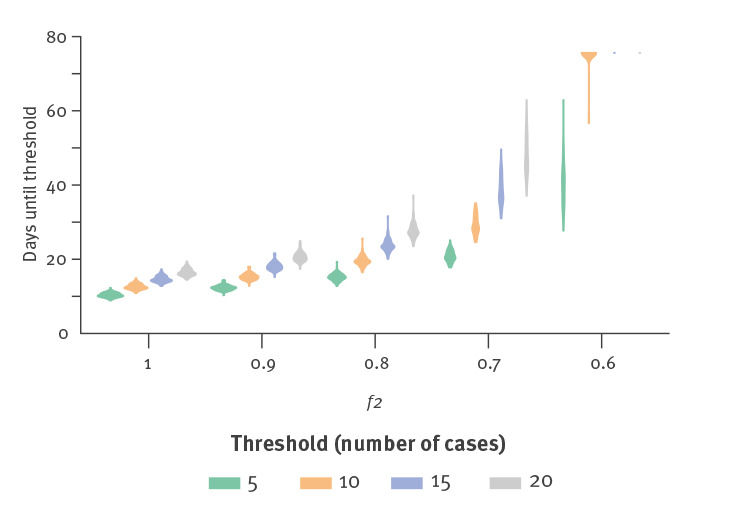
The size of the threshold used to define a substantial difference impacts the time until such a difference occurs, as does the severity of the change in the strength of physical distancing *f*, British Columbia, Canada, May–July 2020

The time until a substantial difference arises depends on what is considered to be a substantial difference, and on the underlying uncertainty. We explored the impact of different threshold choices and extents of relaxation measures on time taken to reach a substantial difference (Figure 3). Stronger relaxation of measures (up to baseline contact levels with *f*_2_ = 1) produced a difference quickly, within 10 to 20 days. Similarly, a smaller threshold difference of five cases is reached relatively soon. Overall, it took between 10 and 60 days for a substantial difference to arise, unless the change was small and the threshold was large, in which case our methods may not have found a substantial difference by the end of the simulation period (120 days).

ODE: ordinary differential equation.

The baseline model has *f*_1_ = 0.36 (17 May 2020), and so a change to *f*_2_ = 1 is a complete relaxation of distancing measures, and is detected sooner than a partial relaxation. We varied the threshold for the number of cases of interest in the set {5, 10, 15, 20}. Missing values on the y-axis (for example for values of the active cases level of 0.6) indicate that our methods could not differentiate between the models with and without relaxed physical distancing when the active cases threshold was 15 or 20 cases. For each *f*_2_ and threshold level, we simulated 10,000 ODEs with random initial *R*_0_ values drawn independently from a normal distribution with mean 2.5 and variance 0.15, and for each group of 100 simulated ODEs we computed the days since 17 May 2020 until the excess active cases threshold is detected between the baseline and the condition.

The uncertainty in *R*_0_ and other parameters also impacts the time until there is a substantial difference in trajectories (Supplementary Figure S1). We found that halving uncertainty in the underlying growth rate (by reducing the standard deviation in *R*_0_) reduced the time until detection from between 20 and 40 days to between 12 and 25 days. Uncertainty in other fixed parameters does not have a strong impact on the time (Supplementary Figure S1b) unless the relaxation conditions are weak (*f*_2_ ≤ 0.7). Epidemiological parameters are now well established for COVID-19 [14], but we are less certain of the true value of parameters (*q*, *u_r_*, *u_d_*) that are influenced by behaviours.

We explored the impact of the viral incubation period upon the time until a substantial difference arises, and found little impact (Supplementary Figure S2). This is largely because a few days’ uncertainty in the incubation period is insignificant relative to the overall time scale and threshold of 10 cases to define a substantial difference. In some instances, we could not differentiate between trajectories for the models with distancing and the models without distancing (these are the missing values in the related figures). Also, if the standard deviation in the underlying parameter *R*_0_ is too large, it may not be possible to predict a substantial difference within the time frame of interest.

### Time to detect and quantify a change in distancing measures using reported data

We have conceptually separated the time until there is any substantial difference in model trajectories and the time until we would be able to estimate the impact of a change in distancing using reported case counts. These times may differ as a result of reporting delay, but in some circumstances it may also be possible to observe signals of a change in distancing in reported data before the threshold is met in model projections, due to unmodelled effects or under a lower probability threshold for example.

**Figure 4:**
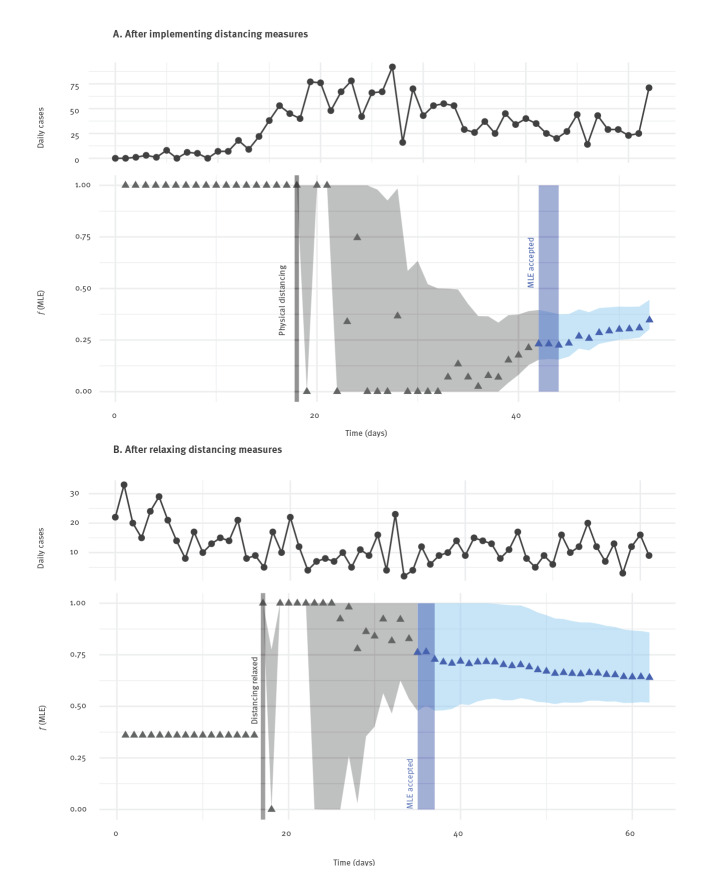
Daily maximum likelihood estimate for the strength of physical distancing using daily reported case count data, British Columbia, Canada, March–July 2020

Figure 4 shows the daily maximum likelihood estimate of the physical distancing parameter *f* over time, in both implementation and relaxation of physical distancing scenarios. We found that it took 26 days to accept the value of *f*_1_ (that is, the MLE changed by less than 5% over a 3-day period, as described in Methods). We estimated *f*_1_ to be 0.22 on the day of acceptance (13 April 2020), but this estimate increased to 0.36 by 22 April, 35 days after distancing and taken to be the end of the observation period. Several larger cluster outbreaks had begun to be observed in BC by this time [15], which may have contributed to this increase in the estimated distancing parameter.

MLE: maximum likelihood estimate.

Grey bands, turning blue after the MLE is accepted, correspond to a 95% credible region obtained from 50 samples of *R*_0_. The reported daily case counts are provided above each figure for reference.

A. Daily maximum likelihood estimate after implementing physical distancing. 26 days to accept the MLE.

B. Daily maximum likelihood estimate after relaxing physical distancing. 20 days to accept the MLE.

In the first few days after the introduction of distancing, we saw considerable noise in the daily MLEs. This is expected, as having only several days of data to inform the estimate means that even small variations in the case counts can have a large effect. The likelihood is flat for a period around the first week after distancing is introduced. The credible intervals are thus more informative than the point estimates. For the introduction of distancing, the time at which the credible interval no longer includes 1.0 may indicate the time at which we are confident that distancing has had a positive effect (even if we cannot yet determine the size of this effect). We were able to observe this over a much shorter time frame: 8 days until the 95% interval drops below *f*_1_ = 0.99 and 11 days until it drops below *f*_1_ = 0.9, in contrast to the 26 days to accept the MLE estimate.

In Figure 4B, we considered the relaxation of physical distancing in BC and calculated daily estimates of parameter *f*_2_. We found that it took 20 days to accept the value of *f*_2_, at which time (6 June) we estimated *f*_2_ to be 0.73, decreasing to 0.64 by the end of the observation period 45 days after distancing: 1 July. We note again the importance of interpreting the daily MLEs in conjunction with the credible intervals, since fluctuations in daily reported cases immediately after a change in distancing measures will have a large impact on the MLE. We also performed the same analysis with simulated data in place of observed case counts, under *f*_2_ values 0.5, 0.65 and 0.9 and assuming that the observation noise and delay remain as pre-relaxation during March and April in BC (Supplementary Figure S3). We estimated that it would take 30 (*f*_2_ = 0.5) or 23 (*f*_2_ = 0.65 or 0.9) days from initiating relaxation of distancing until accepting the MLE *f ^n^*_MLE_.

### Comparison with time-dependent reproductive number *R_t_*

For comparison, we estimated the time-dependent reproductive number [16] (*R_t_*) directly from reported cases, using an assumed serial interval of 5 (standard deviation: 1) days (Supplementary Figure S4). The 95% quantile for *R_t_* dropped below unity 14 days after distancing was introduced (1 April 2020), and it took 18, 24 days after distancing was relaxed for *R_t_’*s 95% quantile to be above unity for *f*_2_ = 0.9 and 0.65, respectively. Even with weekly smoothing, the *R_t_* values fluctuated greatly, particularly after relaxation of distancing. In contrast, the estimates of *f*_1_ and *f*_2_ did not. However, they are not directly comparable to *R_t_* because we estimated a single *f* value over an extended time period, whereas *R_t_* is a daily (smoothed) value. Our model also allows explicit exploration of the effects of delays in reporting and dispersion in case counts (for example from inconsistencies in testing or the impact of spatial spread), which this simple approach for estimating *R_t_* does not.

### Impact of noise and delay in reported case counts

**Figure 5:**
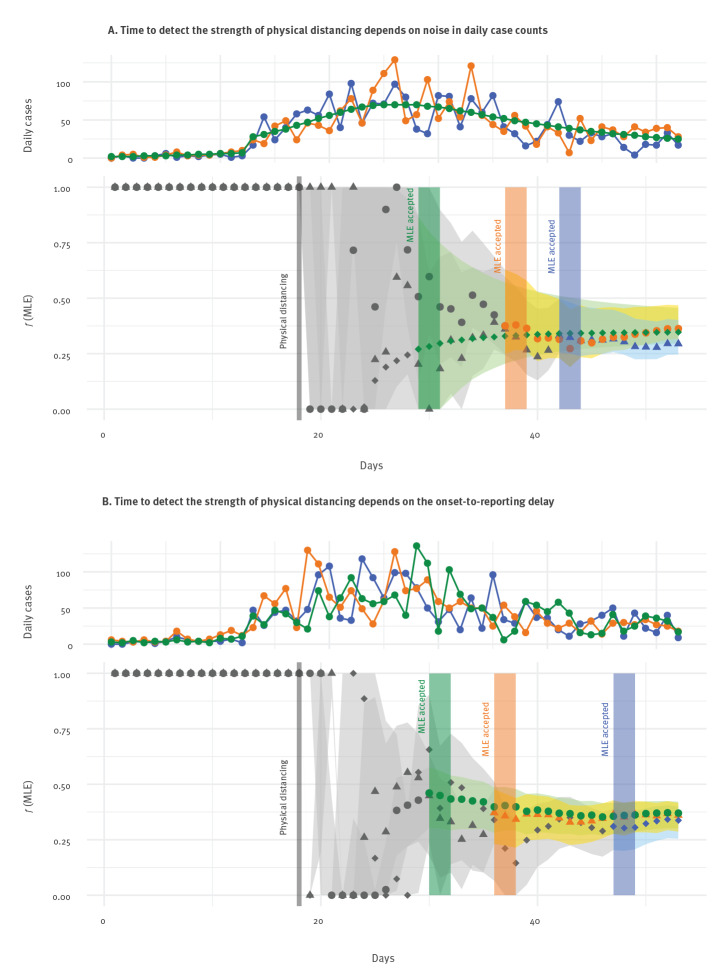
The time to detect the strength of distancing depends on noise in daily case counts and on the onset-to-reporting delay

The time to observe the strength of distancing is impacted by both the level of noise in the case counts and the delay between symptom onset and reporting. We explored the effects of this in Figure 5. As the noise in the daily case counts is reduced (Figure 5A), so is the time to detect the strength of distancing. Under a level of noise realistic for observed case counts in BC (observation dispersion φ = 5), we estimated 26 days to accept *f ^n^*_MLE_. When φ was doubled (corresponding to halving the variance, approximately, when on the order of 50 cases are observed per day), this reduced to 21 days. Under the most optimistic scenario, where there was no noise but still an average 8.7-day delay, the time to accept was 13 days.

MLE: maximum likelihood estimate.

Physical distancing implemented on day 18 at strength *f*_1_ = 0.36. 95% credible band produced from 50 samples of *R*_0_ is shown, and MLE accepted window is highlighted with vertical bars. Results are coloured grey before the MLE is accepted. Simulated daily case counts are provided for reference (top panels).

A. We varied the observation dispersion parameter φ for the daily case counts. Blue: φ = 5, 26 days to accept MLE. Orange: φ = 10, 21 days. Green: no noise, 13 days.

B. We varied the onset-to-reporting delay (mean, variance). Blue: (8.78, 27.4), 31 days to accept. Orange: (4.43, 5.37), 20 days. Green: (0.95, 0.01), 14 days.

A longer delay between symptom onset and case reporting also results in a longer time taken to estimate the strength of distancing (Figure 5B). With a Weibull distributed delay of shape 1.73 and scale 9.85 (mean: 8.78; variance: 27.4) and φ = 5, as estimated for BC in [1], we estimated 31 days to accept the MLE of *f*_1_. When the delay was reduced to shape 2 and scale 5 (mean: 4.43; variance: 5.37), the time to accept was 20 days. Lastly, with a delay shape 10 and scale 1 (mean: 0.95; variance: 0.013), the time to accept was 14 days.

## Discussion

As jurisdictions begin to ease or reimplement physical distancing measures, we must understand how long it may take to observe a statistically significant difference in reported case counts. We found that it generally takes between 10 and 70 days before changes in distancing have a substantial and detectable impact on the underlying model trajectory, depending on the level of parameter uncertainty and the degree of distancing change. In certain cases, we found that these methods are unable to differentiate between the scenarios with relaxed physical distancing and strict physical distancing; for example, if the deviation in *R*_0_ is high and the relative change in physical distancing is low. However, through computing daily estimates of the parameter controlling strength of physical distancing in our model we found that, at least under public health systems in BC, the time taken to detect changes in distancing was 3–4 weeks. Halving the case count dispersion or the mean onset-to-reporting delay in BC during March and April 2020 could have reduced the time taken to understand the strength of physical distancing by ca 20% and 35%, respectively. This highlights the benefit of improved, consistent surveillance systems, and perhaps contact tracing apps if they are able to minimise delays, weekly patterns, or discrepancies in case reporting. Dispersion in observed cases will also occur when spread is inconsistent spatially as well as temporally, for example local clusters or outbreaks. Although spatial spread was not modelled explicitly in this work, we can observe the effects of it through dispersion in daily case counts.

Our analysis has a number of limitations. Although policy changes happen at defined times, if distancing behaviour and other COVID-19 control practices do not change instantaneously then the time frame to detect changes may become longer than we have estimated. We have not explored a wide range of growth rates or baseline prevalence levels and these may affect the results. The transmission model used was a deterministic SEIR variant and did not include stochastic effects (except in the observation model), or age or risk structure. The model includes a fraction of the population practising physical distancing, thereby reducing their contact rates, but does not otherwise include heterogeneity in contact patterns. In particular, we focused on the delay between case onset and reporting, but certain high-risk groups such as healthcare workers may be more likely to get tested and have expedited testing available. Our methodology could readily be extended to structured models, but this requires stratified data and knowledge of mixing patterns across those strata. Indeed, any disease model will include exponential growth and decay; this work is somewhat model-agnostic in that, whatever level of detail goes into producing this exponential behaviour, we can still perform the same eventual inference.

Our approach to determine when the effect of modifying measures is observable relies on using case count data as the indicator for increased community-based transmission. However, public health officials may find outbreaks, even where they do not contribute to statistically higher case counts, by noting epidemiological links among cases (e.g. links through workplace, family, healthcare or gatherings). Changes in these smaller outbreaks may be detected much faster than our 3–4 week estimates, but it remains the case that measures directed towards the general population are the main intervention for COVID-19. Our results focus on estimating impacts on this broader population level and this has long time scales. Sentinel surveillance systems, contact tracing and outbreak detection are among the tools used by public health agencies to gather rapid and more detailed information than case counts during a disease outbreak. These form multifaceted surveillance networks including hospitals, primary care and symptom trackers, which may often be faster than confirmed and laboratory-tested cases. However, these networks also have complex limitations and vary greatly by jurisdiction. Their data are not always consistently or widely published to those outside of public health decision-making. Confirmed case counts remain an informative and comparable source for population-level understanding of COVID-19 control, particularly for modellers seeking a broad assessment of COVID-19 in multiple jurisdictions to compare policy on borders and travel or effectiveness of control measures.

While we focused on the first implementation and relaxation of physical distancing measures for COVID-19, our model can also be used to detect the first time one would expect to see a change in reported case counts in response to modifications of any NPI. For example, we could use this model to explore the effects of introducing digital contact tracing or improved testing for severe acute respiratory syndrome coronavirus 2 (SARS-CoV-2). Our model may also be applied to any region or country with case reporting to determine the relevant time lags, and as such may be used to distinguish the international, national, provincial or regional scales of such effects. For example, SEIR-type models are used to forecast COVID-19 elsewhere e.g. in all 50 states in the United States with the ‘Covid Act Now’ project [17]: each state is associated with a COVID-19 risk level based on how soon their projections arrive at certain constant thresholds on measures such as case counts and intensive care unit headroom used. Our methods could support such regional work by allowing the parameters and baselines to be calibrated, reflecting population density, testing protocols, demographics and cultural factors regarding social contact. The times to observe the impacts of changes in control measures are likely to be region-specific.

We found that the time to detection for a return to widespread transmission owing to relaxed physical distancing measures can be long, indeed considerably longer than the mean incubation period [14,18] or the often used 14-day time period [19-21]. Policymakers need to ensure they have observed the impact of changes in distancing measures before assessing the effect of such changes. In order to decrease the time to detection, we need less noisy testing and faster ways to monitor community transmission. Outbreaks within communities or other sporadic super-spreading events contribute both to ‘noise’ in case counts and to uncertainty in R_0_, particularly if they reveal areas of previous underdetection. It is therefore also important to maintain consistent spatial and temporal surveillance.

Other surveillance techniques could facilitate faster and smoother case detection but suffer from their own limitations. Consistent sentinel surveillance e.g. as are seen in influenza-like illness data, may have a slightly longer delay, but ultimately less noise. Symptom tracker apps could show changes in incidence sooner than laboratory-confirmed case counts, but could suffer from false positive results and may be affected by coverage and usage limitations. Digital contact tracing may also support rapid case finding but will ultimately rely on testing for confirmation. However, case confirmation delays in a contact tracing context are often considerably shorter than in symptom-based testing [22]. Recent research has also investigated detection of SARS-CoV-2 in wastewater [23,24], which, although potentially not revealing of individual-level infection, may provide an early warning system. Similarly, investigation of live mobility data during the disease outbreak may reveal changes in population behaviour, even if such work requires some assumptions about the link to changes in incidence [25,26].

### Conclusion

Given the long time frames to detect changes in COVID-19 measures from case count data, the development of robust combinations of diverse surveillance systems is urgent. For those seeking an overview of COVID-19 trajectories without reference to multifaceted local surveillance data, perhaps at the national level or to support decisions about travel to and from other jurisdictions, it is important not to over-interpret short-term fluctuations in reported case counts.

## Supplementary Data

306000 Supplementary Material
