# Supplementary material for: Long time frames to detect the impact of changing COVID-19 measures, Canada, March to July 2020
Source: Euro Surveill. 2021 Oct 7;26(40):2001204. doi: 10.2807/1560-7917.ES.2021.26.40.2001204 (PMC8511756; doi:10.2807/1560-7917.ES.2021.26.40.2001204)
Supplement: Supplementary Material [file 20-01204_STOCKDALE_Supplementary_material.pdf]

S1. Supplementary methods and results

This supplementary material is hosted by *Eurosurveillance* as supporting information alongside the article “Long time frames to detect the impact of changing COVID-19 measures, Canada, March to July 2020” on behalf of the authors who remain responsible for the accuracy and appropriateness of the content. The same standards for ethics, copyright, attributions and permissions as for the article apply. Supplements are not edited by Eurosurveillance and the journal is not responsible for the maintenance of any links or email addresses provided therein.

Transmission Model

Here we provide full details of the SEIR-type transmission model introduced in Methods. The compartmental model was used in the same form as it was first developed in [1], but we modelled the strength of physical distancing $f(t)$ differently for the purposes of this work.

Upon infectious contact, individuals move from a susceptible state (S) to an exposed state (E_1). From here, the disease progresses through the infectious, pre-symptomatic state (E_2), to infectious and symptomatic (I), and finally to removed (R). Removed individuals are assumed to be not susceptible. The model furthermore incorporates a possible quarantined state (Q), in which individuals are still infected but are unable to infect others. For each state, there is a corresponding “distanced” state which models those individuals performing physical distancing. Individuals in all compartments are able to transition between distancing and non-distancing states, at rates u_d (to distancing) and u_r (from distancing). In practice, the model settles on a fraction $e = u_d/(u_d + u_r)$ of individuals participating in distancing, and it is this quantity which we can more easily interpret and consider as impacting the model dynamics. Survey data from British Columbia during March 2020 [2] suggested that significantly more than 50% of individuals were participating in physical distancing, which motivated our choice of $u_r < u_d$, as found in Table S1. We set $u_r = 0.4$ and $u_d = 1.0$, which leads to a fraction $e = 0.71$ of distancing individuals, a conservative estimate in line with the survey findings in [2]. Note that the quarantined states Q and Q_d in the compartmental model are not particularly impactful for this work, but we included them for consistency with [1], particularly given we used several parameter values estimated in that study.

The differential equations for the non-distancing model compartments are:

$$\begin{aligned}
\frac{\mathrm{d}S}{\mathrm{d}t} &= -\beta\left[I + E_2 + f(I_\mathrm{d} + E_{2\mathrm{d}})\right]\frac{S}{N} - u_\mathrm{d}S + u_r S_\mathrm{d} \\
\frac{\mathrm{d}E_1}{\mathrm{d}t} &= \beta\left[I + E_2 + f(I_d + E_{2\mathrm{d}})\right]\frac{S}{N} - k_1E_1 - u_\mathrm{d}E_1 + u_r E_{1\mathrm{d}} \\
\frac{\mathrm{d}E_2}{\mathrm{d}t} &= k_1E_1 - k_2E_2 - u_\mathrm{d}E_2 + u_r E_{2\mathrm{d}} \\
\frac{\mathrm{d}I}{\mathrm{d}t} &= k_2E_2 - qI - \gamma I - u_\mathrm{d}I + u_r I_\mathrm{d} \\
\frac{\mathrm{d}Q}{\mathrm{d}t} &= qI - \gamma Q - u_\mathrm{d}Q + u_r Q_\mathrm{d} \\
\frac{\mathrm{d}R}{\mathrm{d}t} &= \gamma I + \gamma Q - u_\mathrm{d}R + u_r R_\mathrm{d}.
\end{aligned} \tag{S1}$$

The differential equations for individuals practising physical distancing are analogous:

$$\begin{aligned}
\frac{\mathrm{d}S_\mathrm{d}}{\mathrm{d}t} &= -f\beta\left[I + E_2 + f(I_\mathrm{d} + E_{2\mathrm{d}})\right]\frac{S_\mathrm{d}}{N} + u_\mathrm{d}S - u_r S_\mathrm{d} \\
\frac{\mathrm{d}E_{1\mathrm{d}}}{\mathrm{d}t} &= \ f\beta\left[I + E_2 + f(I_d + E_{2\mathrm{d}})\right]\frac{S_\mathrm{d}}{N} - k_1E_{1\mathrm{d}} + u_\mathrm{d}E_1 - u_r E_{1\mathrm{d}} \\
\frac{\mathrm{d}E_{2\mathrm{d}}}{\mathrm{d}t} &= k_1E_{1\mathrm{d}} - k_2E_{2\mathrm{d}} + u_\mathrm{d}E_2 - u_r E_{2\mathrm{d}} \\
\frac{\mathrm{d}I_\mathrm{d}}{\mathrm{d}t} &= k_2E_{2\mathrm{d}} - qI_\mathrm{d} - \gamma I_\mathrm{d} + u_\mathrm{d}I - u_r I_\mathrm{d} \\
\frac{\mathrm{d}Q_\mathrm{d}}{\mathrm{d}t} &= qI_\mathrm{d} - \gamma Q_\mathrm{d} + u_\mathrm{d}Q - u_r Q_\mathrm{d} \\
\frac{\mathrm{d}R_\mathrm{d}}{\mathrm{d}t} &= \gamma I_\mathrm{d} + \gamma Q_\mathrm{d} + u_\mathrm{d}R - u_r R_\mathrm{d}.
\end{aligned} \tag{S2}$$

In these equations, the strength of physical distancing is represented by the parameter $0 \leq f(t) \leq 1$, with $f(t) = 1$ indicating no physical distancing. This parameter modulates transmission from within the distancing compartments by infected distancing individuals contributing a reduced portion of the force of infection. In addition, individuals in the distancing compartments are less likely to be encountered by others, and so are less likely to become infected themselves. The value of f may be allowed to vary in time as physical distancing is strengthened or relaxed in the population, and in particular, in this work we defined:

$$f(t) = \begin{cases} f_0 & \text{before distancing enacted} \\ f_1 & \text{when distancing measures are in place} \\ f_2 & \text{after some relaxation of distancing.} \end{cases}$$

Recall also the mean number of new cases reported on day t, μ_t:

$$\mu_t = \psi_t \int_0^M k_2(E_2(t-s) + E_{2d}(t-s))w_c(s)\mathrm{d}s.$$

Given a set of daily case count data $\{C_t\}$, where C_t is the number of cases identified on day t, we used a negative binomial likelihood with mean μ_t and dispersion parameter ϕ, $\mathrm{NB}(C_t \mid \mu_t, \phi)$ as in [1], to write the likelihood of the data given the model parameters accounting for dispersion in observation:

$$L(\{C_t\}|R_0, \mathbf{f}, \phi, \boldsymbol{\theta}) = \prod_{t'=1}^{T} \mathrm{NB}(C_{t'} \mid \mu_{t'}, \phi).$$

Here T is the final day for which case counts are available (or, of concern), $\mathbf{f} = (f_0, f_1, f_2)$, and $\boldsymbol{\theta} = (u_\mathrm{d}, u_r, q, \gamma, k_1, k_2, \psi_t)$ are the remaining model parameters, which were assumed to be fixed in all of our experiments except for the simulations in Figure S1. In this parameterization of the negative binomial distribution, the variance scales with the mean according to the dispersion parameter ϕ: Var$[\{C_t\}] = \mu + \mu^2/\phi$. Values of the model parameters for our analysis using British Columbia case count data are provided in Table S1.

Table S1: **Model parameter values for British Columbia**. Values of ψ_t and R_0 were estimated using our model with pre-distancing BC data.

Parameter	Definition	Value
N	Population size	$5,100,000$ [3]
u_d	Rate of moving to physical distancing	1
u_r	Rate of returning from physical distancing	0.4
$1/k_1$	Length of noninfectious exposure period (E_1 to E_2)	5 days [4, 5, 6]
$1/k_2$	Length of pre-symptomatic infectious period (E_2 to I)	1 day [5, 6]
$1/\gamma$	Mean infectious period duration	5 days [7, 8]
q	Quarantine rate	0.05 [9]
ψ_t	Fraction of cases on day t that are tested and reported	0.35 (pre-March 14th)
		0.68 (post-March 14th)
R_0	(Baseline) basic reproduction number	2.57
w_c, shape	Weibull parameter in delay-to-reporting distribution	1.73 [1]
w_c, scale	Weibull parameter in delay-to-reporting distribution	9.85 [1]
ϕ	Observation dispersion parameter of case count distribution	5 [1]

Time to pairwise model divergence

In predictive investigations of the effects of relaxing physical distancing in the main text, we simulated the SEIR-type model described above forwards in time, from 1 May to 1 July. We investigated situations in which the amount of physical distancing was modulated on 17 May through a forcing

of the ODE parameter f_2, and in which many of the ODE parameters were modulated through grid-searches. We also considered the introduction of physical distancing in British Columbia, assuming that physical distancing was introduced 18 March by modulation of parameter f from $f_0 = 1$ to $f_1 = 0.4$ and $f_1 = 0.7$, and compared to a baseline model in which f remained at 1. We investigated confidence intervals around the number of active case in early/mid-April, by considering 100 replicates in the ODE simulations, each with a value of R_0 drawn independently from the prior, with standard deviation 0.15.

In Results, we provided evidence that the physical distancing undertaken by people in British Columbia corresponded to a modeled value for f_1 of 0.36. In the relaxation of distancing scenario, we therefore considered a baseline model in which the value of f remained at 0.36 for the duration of the time. We considered alternative relaxation models in which the value of f changed from $f_1 = 0.36$ to either $f_2 = 0.5$ (weak relaxation), $f_2 = 0.65$ (medium relaxation) or $f_2 = 0.9$ (strong relaxation) on 17 May. For each condition, we again considered 100 replicates each with a value of R_0 drawn from the prior.

Impact of uncertainty in model parameters

We considered two experiments wherein the uncertainty about the ODE parameters was modulated, and recorded the time to detect a threshold of excess in the active cases above baseline models. In our first experiment we focused on R_0. In the main text, we considered ODEs in which the R_0 parameter was initialized to a draw from a normal distribution with mean 2.57 and standard deviation 0.15. This reflects our broad understanding about the uncertainty in R_0. In all conditions, the baseline indicated a situation in which physical distancing is not relaxed, and remains in effect. If the standard deviation is varied away from 0.15, then the days until the threshold is reached (*i.e.*, a significant difference between the baseline and a relaxed condition is seen) is affected. The results of varying the standard deviation between 0.01 and 0.4 (considering the values in the set {0.01, 0.11, 0.21, 0.31, 0.41}) are shown in Figure S1a. In that Figure, we see that the days until a significant increase in the active cases can vary from 10 to 50 days. Missing values in the lines in this plot indicate that our methods could not determine whether or not the condition will ever differ from the baseline: if the standard deviation of R_0 is > 0.11, we do not predict that a weak relaxation of physical distancing to $f_2 = 0.6$ will result in a substantial increase in active cases. However, if the standard deviation of R_0 is < 0.11 a relaxation of physical distancing to $f_2 = 0.6$ could lead to a significant increase in active case count after 20 or 40 days. This is not because the change itself is different, but is a consequence of uncertainty in the baseline.

We also considered variation of the ODE parameters $1/\gamma$, k_1, k_2, q, u_d and u_r. We varied each of these parameters around their default values (given in Table S1) by $X\%$, with X varied in the

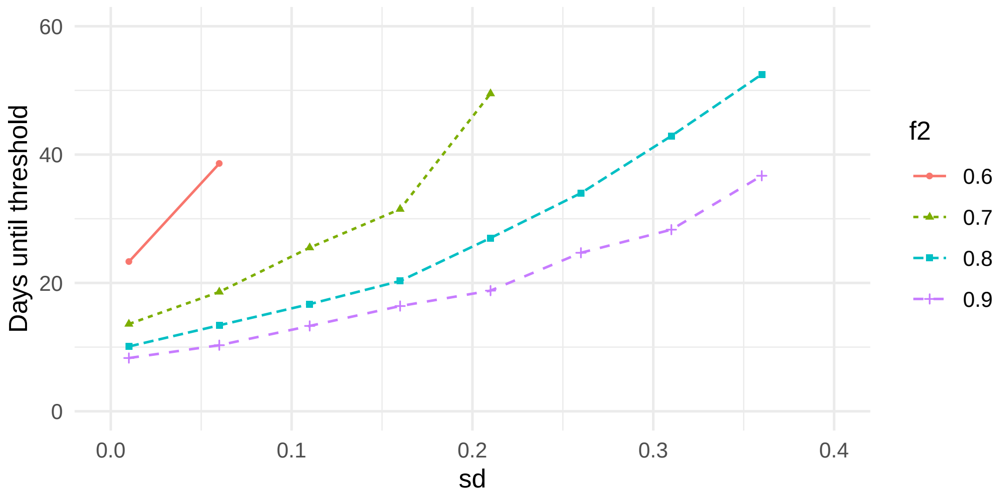

(a)

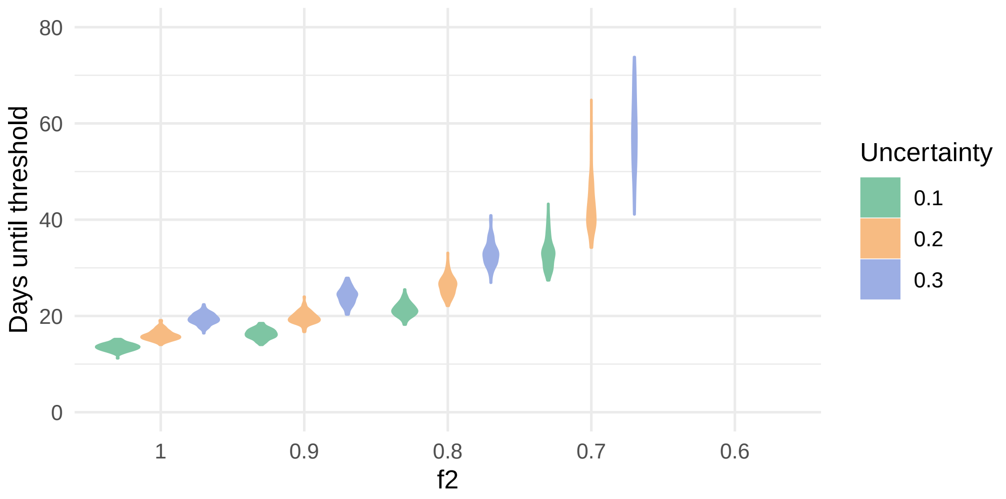

(b)

Figure S1: **Impact of uncertainty in the model parameters on the time to exceed the active case threshold.** (a) Days until active cases in the relaxed model exceed the baseline model (y-axis), with varying levels of the standard deviation for R_0 (*sd*, x-axis). There are four relaxation levels (all starting relaxation on 17 May). Parameter settings are identical to other figures aside from *sd*. The missing values indicate that our methods could not differentiate between the models. (b) The variables $1/\gamma$, k_1, k_2, q, u_d and u_r were all varied between 10% and 30% around their fitted values (with the three levels described in the legend). The value that f was relaxed to is shown in the x-axis (before relaxation, f_1 was set to 0.36). So, R_0 was sampled at ODE initialization, but so too now were the 6 variables mentioned (with independent draws from the uniform distribution). For each f_2 level, 100 iterations were considered each with 100 replicates. For $f_2 = 0.6$, we did not observe enough difference between the model trajectories over the period observed (120 days) to determine the active case threshold.

set $\{10, 20, 30\}$. For each X (uncertainty) level, we considered simulations in which the ODE was initialized with random parameters. For the basic reproduction number, R_0 was initialized as a normal distribution with mean 2.57 and variance 0.15 (as is done in the main text), and for the parameters $1/\gamma$, k_1, k_2, q, u_d and u_r we initialized each parameter by drawing from a uniform distribution. For each parameter, the range of the uniform distribution had a minimum value of the 'default' value of that parameter (given by Table S1) minus $X\%$ of the default value, and maximum value of the 'default' plus $X\%$. We then recorded the days until an active case count excess of 10 was obtained in 95% of the simulations. The number of iterations and the number of replicates was identical to Figure 3 (described in the main text). Density plots for the number of days until the threshold on the excess of active case counts are shown in Figure S1b for a variety of relaxation levels. These results show that if the uncertainty about the parameters of our SEIR-type model is 10%, then relaxing distancing totally $f_2 = 1$ can result in 10-15 days before the threshold of 10 excess cases is reached. This increases slightly to 15-20 and 17-22 days if the uncertainty is increased to 20% or 30%. In addition, if physical distancing is relaxed more moderately (to $f_2 = 0.7$ for example), then it may be around 70 days before an excess of 10 active cases is seen, given moderate uncertainty in the model parameters. For relaxation to $f_2 = 0.6$, we did not observe enough difference between the model trajectories to determine the active case threshold. This may be due to either a) a significant difference in the trajectories only occurred after the 120 day simulation window considered here or b) one model's active cases never become significantly larger than the others a significant percent of the time, given the uncertainty in the model parameters.

The above results indicate that, for smaller or moderate changes in f, uncertainty about the model parameters can substantially increase the number of days until definitive statements about the effects of relaxing physical distancing can be made. For larger changes in f this is less impactful, with the total range of the number of days until effects are seen varying between 10 and 75.

Effect of the incubation period

We varied the incubation period to explore the impact on the time before the active cases threshold of 10 is reached. The incubation period was defined as $1/k_1 + 1/k_2$, with default values $k_1 = 1/5$ and $k_2 = 1$ throughout this work. We varied the incubation period between 6 and 10 days by modulating k_1 and k_2 appropriately (with the same scaling factor for each) and show the results in Figure S2. For each incubation period and relaxation f_2 level, we performed 50 replicates and plotted the median number of days until the active cases threshold is reached. We found that there is not much variation in the days to threshold as the incubation period is varied. This suggests that

the variance in active case due to uncertainty about R_0 may overwhelm variation in the incubation period, over the range considered.

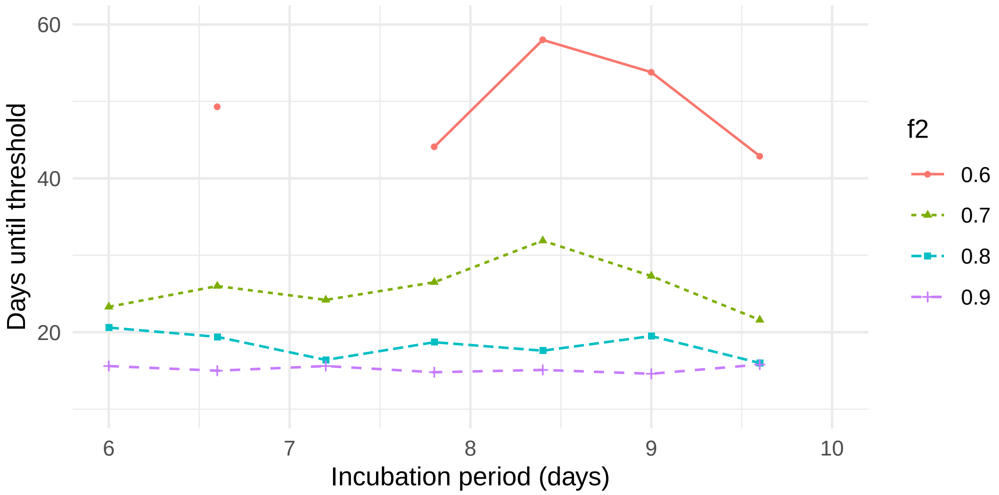

Figure S2: **The incubation period has a limited effect on the time before a substantial difference occurs between two simulated scenarios**. The initial value f_1 is 0.36. The curves were obtained under the uncertainty assumption that R_0 is normally distributed with a mean 2.57, and a standard deviation 0.15. The incubation period is $1/k_1 + 1/k_2$. For each setting of the incubation period, we rescaled k_1 and k_2 evenly from their fitted values ($k_1 = 1$ and $k_2 = 1/5$) to achieve the incubation period in days (*e.g.*, for an incubation period of 7 days $1/k_1 + 1/k_2 = 7$, and so the values for k_1 and k_2 used were $1 * (6/7)$ and $1/5 * (6/7)$ respectively). Missing values indicate that our methods could not distinguish between simulations in which physical distancing was relaxed and simulations in which physical distancing was not relaxed (indicating that a relaxation of f_2 to 0.6 may not have an effect for much of the range of incubation periods considered)

Daily MLE using simulated data

In addition to the experiments in the main manuscript which estimated daily MLEs of f_1 or f_2 using reported case counts from British Columbia, we performed a simulation study in which we estimated daily MLEs of f_2 using simulated data on the relaxation of physical distancing to a number of fixed f_2 values (0.5 0.65 and 0.9). The benefit of using simulated data is that we can be confident the change in distancing occurred exactly on the date stated. We set the observation noise and delay as estimated pre-relaxation during March/April in BC ($\phi = 5$, onset-to-reporting delay was Weibull with mean 8.78, variance 27.4), both in the simulation of data and in the daily MLE analysis. The results of this are shown in Figure S3. We found similar time frames to identify the strength of physical distancing as with the observed data: 30 days for $f_2 = 0.5$, and 23 days for both $f_2 = 0.65$ and $f_2 = 0.9$. Smaller changes in distancing take longer time frames to identify.

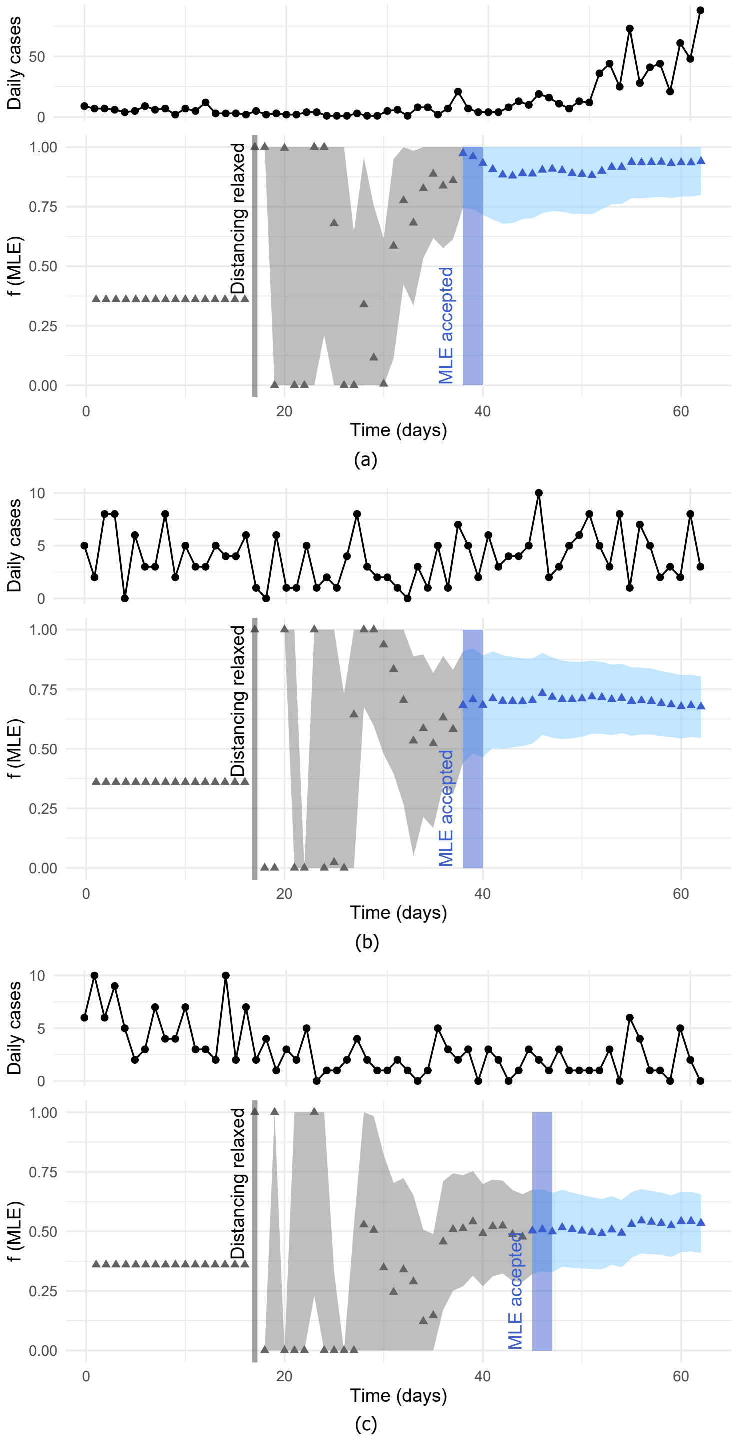

Figure S3: **Daily maximum likelihood estimate f^n_{MLE} for the strength of physical distancing after distancing has been relaxed, f_2, in BC, Canada using simulated data.** (a) $f_2 = 0.9$ (23 days to accept MLE), (b) $f_2 = 0.65$ (23 days to accept MLE), and (c) $f_2 = 0.5$ (30 days to accept MLE). Grey bands, turning blue after the MLE is accepted, correspond to an estimated 95% credible region, obtained from 50 samples of R_0. The simulated daily case counts are provided above each figure for reference.

Estimation of R_t

For comparison with the daily MLE method applied to British Columbia case count data in Figures 4 and S3, we also performed estimation of R_t using the *EpiEstim* package [10] in *R*. Results are shown in Figure S4. Note that *EpiEstim* assumes that all cases from the first time step are imported cases, leading to inflated R_t estimates during the first week. To account for this, when finding the earliest time that R_t was less than/greater than 1.0 at the 95% level, we ignored this first week of estimation.

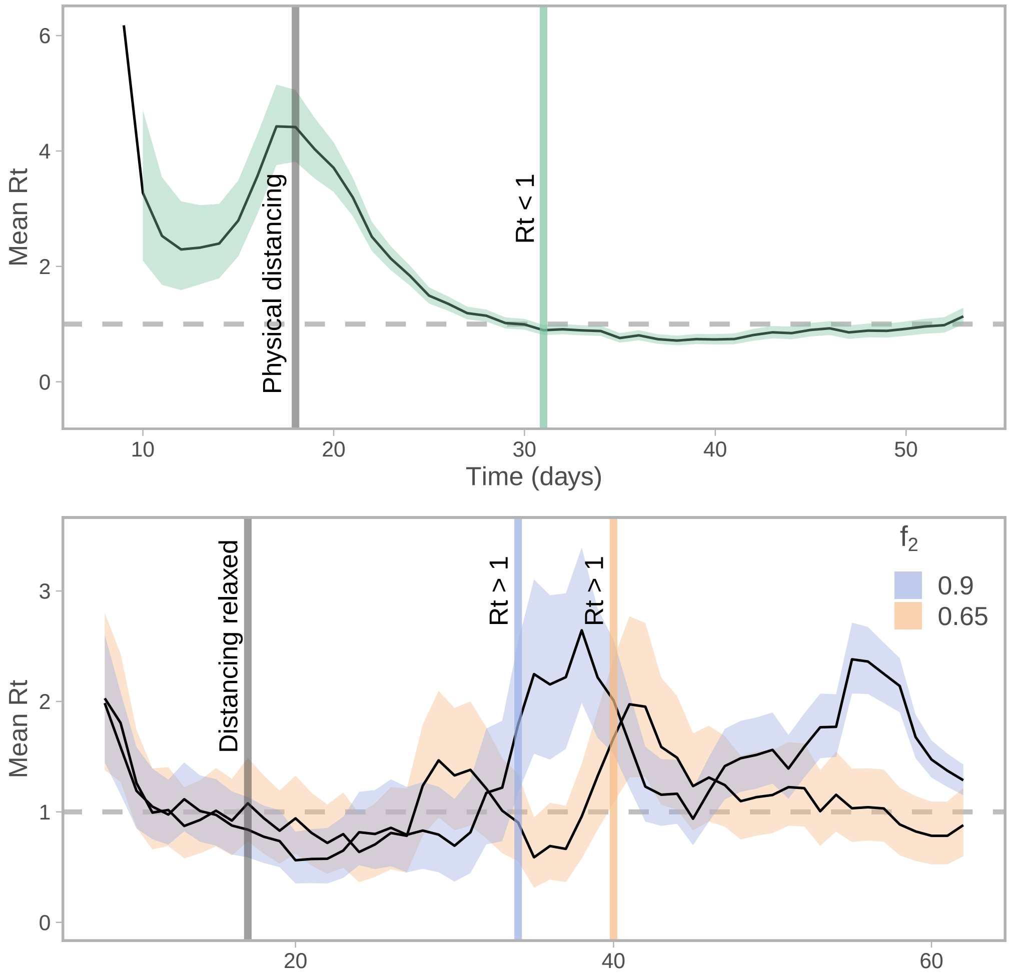

Figure S4: **Median estimates of the time-dependent reproduction number R_t in British Columbia, using a weekly sliding window and assumed serial interval of 5 (sd 1) days**. Top panel: after implementing distancing, using true observed case counts (14 days until $R_t < 1$ at the 95% level). Bottom panel: after relaxing distancing, using simulated data (18, 25 days until $R_t > 1$ at the 95% level for $f_2 = 0.9, 0.65$, respectively), under the assumption that $f_1 = 0.36$ and observation noise and delay remained as pre-relaxation during March/April in BC. Coloured bands correspond to 95% quantiles.

References

[1] Anderson SC, Edwards AM, Yerlanov M, Mulberry N, Stockdale JE, Iyaniwura SA, et al. Quantifying the impact of COVID-19 control measures using a Bayesian model of physical distancing. PLoS computational biology. 2020;16(12):e1008274.

[2] Korzinski D, Kurl S. COVID-19 Carelessness: Which Canadians say pandemic threat is 'overblown'? And how are they behaving in turn?; 2020. [Online; last accessed December 2020]. Available from: http://angusreid.org/covid-19-serious-vs-overblown/.

[3] The Ministry of Citizens Services. Population estimates—Province of British Columbia, Canada;. Accessed April 1st, 2020. https://www2.gov.bc.ca/gov/content/data/statistics/people-population-community/population/population-estimates.

[4] Li Q, Guan X, Wu P, Wang X, Zhou L, Tong Y, et al. Early transmission dynamics in Wuhan, China, of novel coronavirus-infected pneumonia. New England Journal of Medicine. 2020;382(13).

[5] Tindale LC, Stockdale JE, Coombe M, Garlock ES, Lau WYV, Saraswat M, et al. Evidence for transmission of COVID-19 prior to symptom onset. Elife. 2020;9:e57149.

[6] Ganyani T, Kremer C, Chen D, Torneri A, Faes C, Wallinga J, et al. Estimating the generation interval for coronavirus disease (COVID-19) based on symptom onset data, March 2020. Eurosurveillance. 2020;25(17):2000257.

[7] Kucharski AJ, Russell TW, Diamond C, Liu Y, Edmunds J, Funk S, et al. Early dynamics of transmission and control of COVID-19: A mathematical modelling study. Lancet Infectious Diseases. 2020;20(5).

[8] Zou L, Ruan F, Huang M, Liang L, Huang H, Hong Z, et al. SARS-CoV-2 viral load in upper respiratory specimens of infected patients. New England Journal of Medicine. 2020;382(12).

[9] The Public Health Agency of Canada. Coronavirus disease (COVID-19): Outbreak update;. Accessed April 14th, 2020. https://www.canada.ca/en/public-health/services/diseases/2019-novel-coronavirus-infection.html.

[10] Cori A, Ferguson NM, Fraser C, Cauchemez S. A New Framework and Software to Estimate Time-Varying Reproduction Numbers During Epidemics. American Journal of Epidemiology. 2013;178(9).
